# Comparison of Equivalence between Two Commercially Available S499-Phosphorylated FMRP Antibodies in Mice

**DOI:** 10.1371/journal.pone.0143134

**Published:** 2015-11-18

**Authors:** Conner D. Reynolds, Gregory D. Smith, Taylor S. Jefferson, Joaquin N. Lugo

**Affiliations:** 1 Department of Psychology and Neuroscience, Baylor University, Waco, Texas, United States of America; 2 Institute of Biomedical Sciences, Baylor University, Waco, Texas, United States of America; Naval Research Laboratory, UNITED STATES

## Abstract

Fragile X syndrome (FXS) develops from excessive trinucleotide CGG repeats in the 5’-untranslated region at Xq27.3 of the *Fmr-1* gene, which functionally silences its expression and prevents transcription of its protein. This disorder is the most prominent form of heritable intellectual deficiency, affecting roughly 1 in 5,000 males and 1 in 10,000 females globally. Antibody specificity and selectivity are essential for investigating changes in intracellular protein signaling and phosphorylation status of the Fragile X Mental Retardation Protein (FMRP). Currently, both PhosphoSolutions^®^ and abcam® produce commercially available S499-phosphorylated FMRP specific antibodies. The antibody from PhosphoSolutions^®^ has been validated in previous studies; however, the antibody from abcam^®^ antibody has yet to receive similar validation. This study aims to determine whether these two antibodies are true equivalents through western blot analysis of both *NS-Pten* knockout (KO) and *Fmr-1* KO mice strains. We prepared hippocampal synaptosomal preparations and probed the samples using total FMRP, abcam^®^ phosphorylated FMRP, and PhosphoSolutions^®^ phosphorylated FMRP antibodies. We found that there was a significant increase in phosphorylated FMRP levels using the abcam® and PhosphoSolutions^®^ antibodies in the *NS-Pten* KO mice compared to wildtype mice. However, there was much more variability using the abcam^®^ antibody. Furthermore, there was a band present in the *Fmr-1* KO for the phosphorylated FMRP site using the abcam^®^ antibody for western blotting but not for the PhosphoSolutions^®^ antibody. Our findings strongly suggest that the antibody from abcam^®^ is neither specific nor selective for its advertised targeted substrate, S499-phosphorylated FMRP.

## Introduction

Since the *Fmr-1* gene was discovered in 1991, its product, the Fragile X Mental Retardation Protein (FMRP), has been a topic of major discussion in the investigation of inherited intellectual deficiencies [1]. It is most frequently addressed in Fragile X Syndrome (FXS), the most prominent form of heritable intellectual disability [2]. This disorder develops from excessive trinucleotide CGG repeats in the 5’-untranslated region at Xq27.3 of the *Fmr-1* gene, functionally silencing its expression and preventing transcription of its protein [1]. Fragile X syndrome is observed in approximately 1 in 5,000 males and 1 in 10,000 females globally [3, 4]. Altered FMRP levels have also been observed in disorders unassociated with an expansion in *Fmr-1*, such as schizophrenia, bipolar disorder, major depression, autism spectrum disorder, and epilepsy [5–7]. The involvement of FMRP in such a wide array of disorders results from its role in protein synthesis throughout the brain.

Primarily associating with polyribosomes, FMRP has been shown to negatively regulate protein synthesis via post-transcriptional degradation of target mRNA [8]. Phosphorylation status determines where in the cell FMRP performs this function. Unphosphorylated FMRP tends to associate with actively translating polyribosomes, while phosphorylated FMRP tends to associate with stalled polyribosomes. This protein is preferentially phosphorylated at the highly conserved serine 499 (S499) residue, which subsequently triggers the phosphorylation of nearby serine residues [9]. Thus, in studies investigating the expression of FMRP, discerning its phosphorylation status is imperative.

FMRP-mediated transcriptional repression has been well-characterized in the Phosphoinositide 3-kinase|serine/threonine kinase|mammalian target of rapamycin (PI3K-Akt-mTOR) intracellular signaling pathway. This pathway has essential roles in nutrition, energy metabolism, and cell growth throughout the entire body, with specific roles in neural plasticity, learning, and memory in the brain [10–13]. FMRP is activated downstream of this pathway, subsequently initiating a negative feedback loop and preventing hyperactivation of this pathway. While it is hypothesized to result via FMRP-mediated suppression of the PI3K-enhancer, PIKE-S [14], a definitive mechanism of action still remains unclear.

Phosphatase and tensin homolog (PTEN) also functions as a critical negative regulator of PI3K-mediated conversion of Phosphatidylinositol 4,5-bisphosphate (PIP_2_) to Phosphatidylinositol (3,4,5)-trisphosphate (PIP_3_). Disruption of this protein causes hyperactive PI3K-Akt-mTOR signaling and has been associated with spontaneous seizures, disorganization of neural circuitry, macrocephaly, developmental delay, and autism spectrum disorders [15, 16]. Previous studies have also confirmed this disruption leads to a downstream increase of total FMRP and phosphorylated FMRP in the brain [17, 18].

Protein signaling changes in the brain are most readily visualized using western blot analysis. This method uses primary antibodies that bind with target protein sequences to show relative expression between groups. However, producing antibodies that recognize specific phosphorylated amino-acid residues is a difficult process, potentially leading to target substrate variability [reviewed in [19]]. PhosphoSolutions^®^ and abcam^®^ are two companies that have produced an antibody that recognizes phosphorylated FMRP. The efficacy of PhosphoSolutions^®^ antibody product has been validated in a *Fmr-1* KO mice study [7]. However, the antibody from abcam^®^ has yet to receive any similar validation of efficacy. Therefore, the aim of this study was to compare expression levels observed using the PhosphoSolutions^®^ and abcam^®^ antibody products and to determine whether they yield equivalent results when used in assays. Given the link between PTEN disruption and pFMRP hyperphosphorylation described above, the *NS-Pten* KO mice is an ideal animal model for comparing the equivalency of these antibodies. These antibodies will also be compared using *Fmr-1* KO mice in order to confirm that both antibodies are truly targeting S499-phosphorylated FMRP. The results below provide evidence that the antibody from abcam^®^ is not labeling S499-phosphorylated FMRP, while the antibody from PhosphoSolutions^®^ does label for S499-phosphorylated FMRP.

## Methods and Methods

### Ethics Statement

This study was carried out in strict accordance with the recommendations in the Guide for the Care and Use of Laboratory Animals of the National Institutes of Health. The protocol was approved by Baylor University Institutional Care and Use Committee (Animal Assurance Number A3948-01)

### Animals

Two strains of mice were used in this investigation. Neuron subset-specific *Pten* conditional mice have been previously described in literature as GFAP-*Cre* ^loxP/loxP^ [15, 20]. *NS-Pten* ^+/+^ wild type (WT) and *NS-Pten* ^loxP/loxP^ knockout (KO) mice were produced for this experiment by breeding *NS-Pten* ^loxP/+^ heterozygous parents. *Fmr-1* ^+/+^ wild type (WT) and *Fmr-1* ^-/-^ knockout (KO) mice were produced for this experiment by breeding *Fmr-1* ^-/-^ male and female mice and separate breeding of *Fmr-1* ^+/+^ male and female mice. We originally purchased this strain through The Jackson Laboratory (FVB.129P2-*Pde6b*+ Tyr^c-ch^ Fmr1^tm1Cgr^/J). All mice were subsequently housed at an ambient temperature of 22°C, with a 14-hour light and 10-hour dark diurnal cycle. These mice were also given *ad libitum* access to food and water. All procedures involving mice were conducted in compliance with the National Institute of Health Guidelines for the Care and Use of Laboratory Animals.

### Western Blotting

*NS-Pten* and *Fmr-1* wildtype and knockout mice were sacrificed at approximately 8-weeks of age and hippocampi were rapidly dissected. The resulting samples were then rinsed in 1X phosphate buffer solution, placed on dry ice, and stored at -80°C until used. Hippocampi were homogenized in ice-cold homogenization buffer (0.32M sucrose, 1mM EDTA, 5mM Hepes) containing protease inhibitor cocktail (Sigma, USA) and processed for western blotting as previously described [21]. This procedure produced both crude synaptosomes and total homogenate samples. Only the crude synaptosomes were used in our total analyses of FMRP and phosphorylated FMRP expression. Equivalent protein concentrations were confirmed using the Bradford Protein Assay (Bio Rad, Hercules, CA, USA) and diluted in Laemmli loading buffer (4X: 0.25M Tris, pH 6.8, 6% SDS, 40% Glycerol, 0.04% Bromophenol Blue, 200mM Dithiothreitol). Following SDS-PAGE, proteins were transferred to Hybond-P polyvinyl difluoride membranes (GE Healthcare, Piscataway, NJ, USA). Membranes were then incubated for 1 hour at room temperature in blocking solution [5% non-fat milk diluted in 1X Tris Buffered Saline (50mM Tris-HCl, pH 7.4, 150mM NaCl) with 0.1% Tween (1X TTBS) and 1mM Na_3_VO_4_]. The membranes were then incubated overnight at 4°C with the following primary antibodies in 5% milk in TTBS: FMRP (1:1,000; Cell Signaling Technology, Catalog Number 4317, Boston, MA, USA); pFMRP (1:500 PhosphoSolutions^®^, Catalog Number p1125-499, Aurora, CO, USA); pFMRP (1:500 abcam^®^, Catalog Number ab48127, Cambridge, MA, USA), and actin (1:5,000; Sigma Chemical Co., Catalog Number A2066, USA). After the incubation period membranes were washed in 1X TTBS (3 x 5 min). Membranes were then incubated with horseradish peroxidase labeled secondary antibody, anti-rabbit IgG (1:2,000; Cell Signaling Technology, Boston, MA, USA). Following a final wash cycle in 1X TTBS, membranes were then incubated with GE ECL Prime (GE Healthcare, Piscataway, NJ, USA). Immunoreactive bands were captured by a ProteinSimple western blot imaging system (ProteinSimple, Santa Clara, CA, USA).

### Optical Densitization

ProteinSimple AlphaView software was then used to measure the optical density of resulting immunoreactive bands. Measurements obtained from all bands of interest were normalized to actin levels within the same lane. All experimental points represent a single mouse each (n = 1). All groups were normalized to the average of the control group (NS-*Pten* WT) per blot. All values from WT mice represent biological replicates and were collected from WT littermates.

### Statistical Analysis

The western blotting data were analyzed using an independent samples t-test. All data were analyzed using either GraphPad Prism 6 software (La Jolla, CA) or SPSS 20.0 (IBM, USA). Values are shown as mean ± S.E.M. for each group.

## Results

### *NS-Pten* KO Mice Show Increased Total FMRP Expression

**Fig 1:**
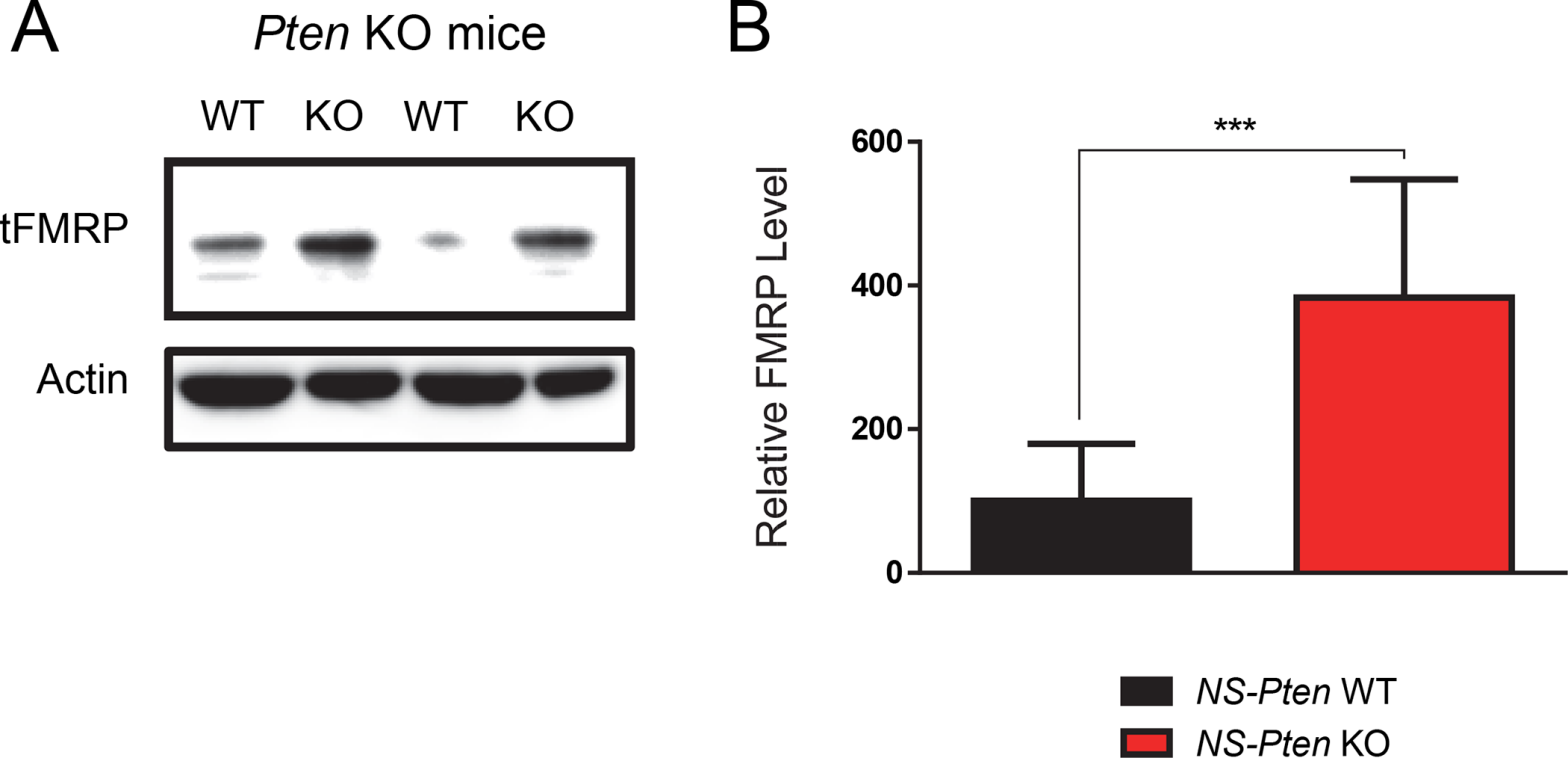
*NS-Pten* KO leads to increased expression of FMRP. Hippocampal tissue from *NS-Pten* wildtype (WT) and knockout (KO) mice were examined for total FMRP levels using western blotting. (Fig 1A) The figure shows a representative blot from two *NS-Pten* WT and KO samples. (Fig 1B) Graphs show the mean (± SEM) of WT and KO mice. *** = p < 0.001. n = 9 per group.

We first confirmed the results of a previous study by Lugo et al. (2014) that indicated *Pten* disruption substantially increases total FMRP (Cell Signaling Technology, Catalog Number 4317, Boston, MA, USA) levels. As expected, differential expression of the protein between groups showed significant increase in total FMRP levels in the *NS-Pten* KO mice compared to WT mice *t*(1,16) = 4.6, *p*<0.001 (n = 9) (Fig 1).

### PhosphoSolutions^®^’ & abcam^®^’s S499-Phosphorylated FMRP Antibodies Both Indicate Increased Expression in *NS-Pten* KO Mice

**Fig 2:**
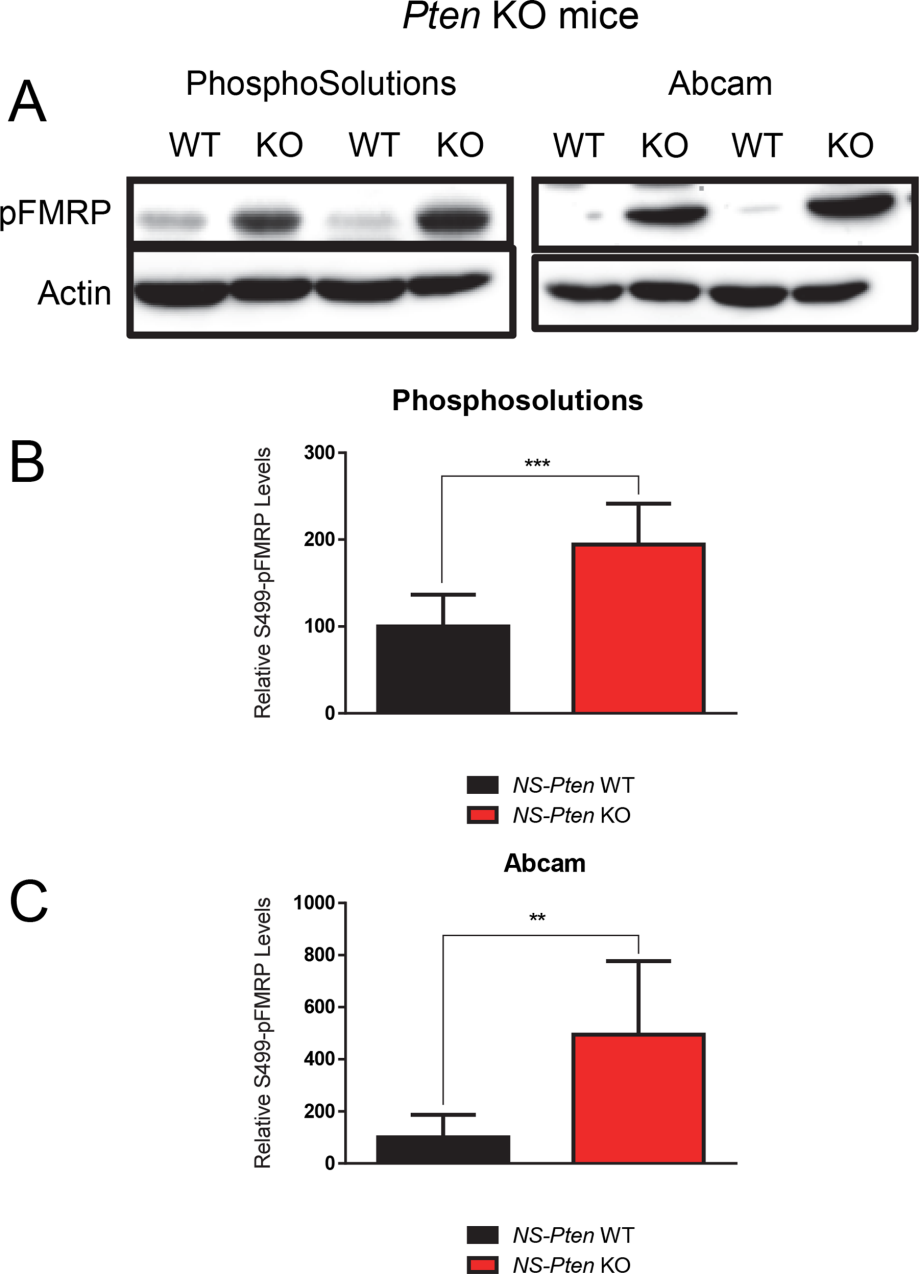
*NS-Pten* KO leads to increased expression of S499-Phosphorylated FMRP. (Fig 2A) S499-phosphorylated FMRP expression indicated by the antibody from PhosphoSolutions^®^ and abcam^®^ for *NS-Pten* wildtype (WT) and knockout (KO) mice. The figures are representative blots from WT and KO hippocampal samples probed for the respective antibodies. (Fig 2B) S499-phosphorylated FMRP expression indicated by the Phosphosolutions^®^ antibody. (Fig 2C) S499-phosphorylated FMRP expression indicated by abcam^®^’s antibody *NS-Pten* for WT and KO mice. Graphs show the mean (± SEM). n = 9 per group. ** = p < 0.01; *** = p < 0.001.

Previous studies have found that disruptions in the PI3K-Akt-mTOR pathway lead to increases in total phosphorylated FMRP in the brain [17, 18]. Because the efficacy of PhosphoSolutions^®^’ antibody has been confirmed in previous studies [7, 22], we used it as a control in examining the influence of *NS-Pten* deletion on pFMRP expression. We found a significant increase in total S499-phosphorylated FMRP levels in the *NS-Pten* KO mice compared to controls *t*(1,16) = 4.76, *p* < 0.001 (n = 9) (Fig 2B). We then used the abcam^®^ antibody to determine how its results compare to those of PhosphoSolutions^®^. We found that it also showed a significant increase in total pFMRP levels in the *NS-Pten* KO mice compared to controls *t*(1,16) = 4.0, *p*<0.01 (n = 9) (Fig 2C). This confirms that both antibody products are reasonably able to detect total S499-phosphorylated FMRP expression.

### PhosphoSolutions^®^’ and abcam^®^’s S499-Phosphorylated FMRP Antibodies Differ in Expression and Variance in *NS-Pten* KO Mice

**Fig 3:**
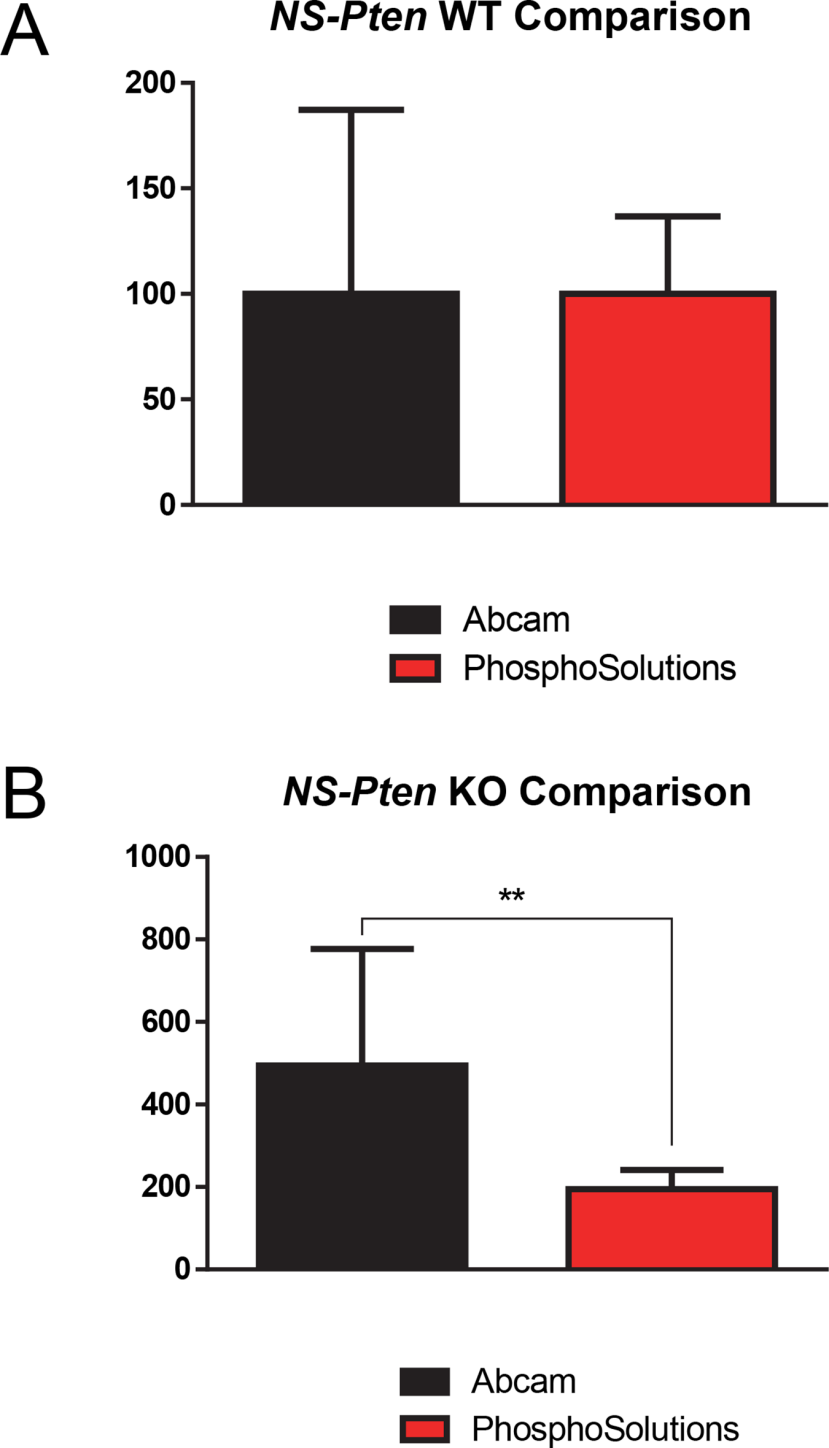
Comparison of S499-Phosphorylated FMRP Expression Between Antibodies. (Fig 3A) Comparison of S499-phosphorylated FMRP expression indicated between the antibodies from PhosphoSolutions^®^ and abcam^®^ in *NS-Pten* WT mice (Fig 3B) Comparison of S499-phosphorylated FMRP expression indicated between PhosphoSolutions^®^ and abcam^®^ antibodies in *NS-Pten* KO mice. ** = p < 0.01. Graphs show the mean (± SEM). n = 9 per group.

We next wanted to determine if there were any differences between the results obtained by PhosphoSolutions^®^ and abcam^®^ antibodies. Comparing the results from both antibodies in *NS-Pten* WT S499-phosphorylated FMRP expression, we found no significant differences *t*(1,16) = 0.01, *p*>0.99 (n = 9) (Fig 3A). However, there were significant differences in variance between the two antibody products *F*(8,8) = 5.67, *p* < .05, with the antibody from abcam^®^ showing greater variance than the antibody from PhosphoSolutions^®^.

In comparing relative S499-phosphorylated FMRP expression in *NS-Pten* KO mice, we found significant differences between the two antibodies *t*(1,8.4) = 3.1, *p*<0.01 (n = 9) (Fig 3B), with the abcam^®^ antibody indicating much greater expression than the PhosphoSolutions^®^ antibody. There were also significant differences in variance between the two antibody products *F*(8,8) = 36.29, *p*<0.001, with the antibody from abcam^®^ showing greater variance than the antibody from PhosphoSolutions^®^.

### Expression Levels Indicated By PhosphoSolutions^®^ and abcam^®^ Antibodies S499-Phosphorylated FMRP Antibodies Show No Correlational Relationship in *NS-Pten* KO Mice

**Fig 4:**
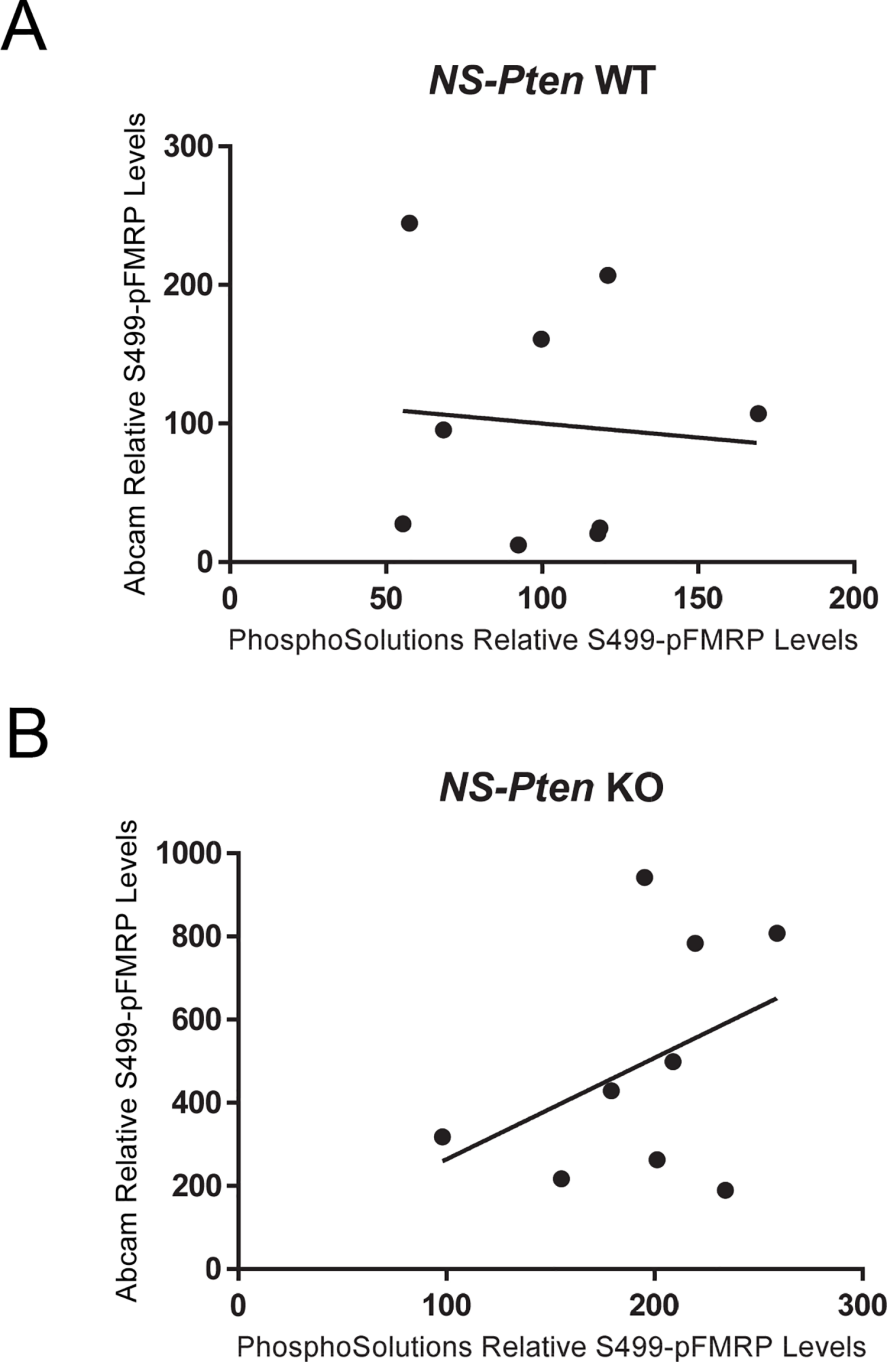
Comparison of S499-Phosphorylated FMRP Expression Between Antibodies. (Fig 4A) Correlation of S499-phosphorylated FMRP expression indicated between PhosphoSolutions^®^ and abcam^®^ antibodies in *NS-Pten* WT mice. (Fig 4B) Correlation of S499-phosphorylated FMRP expression indicated between PhosphoSolutions^®^ and abcam^®^ antibodies in *NS-Pten* KO mice. The line represents the line of best fit. n = 9 per graph.

With the previous differences in indicated expression found between antibodies, we next wanted to determine whether there is a correlational relationship between the S499-phosphorylated FMRP expressions indicated by PhosphoSolutions^®^ and abcam^®^ antibody products. Theoretically, if these antibodies are measuring the same target substrate, then a perfect correlation should exist. In *NS-Pten* WT mice, correlational analysis between the expression levels between antibodies failed to show a significant relationship *r*(16) = -0.09, *p* = 0.83 (Fig 4A). In *NS-Pten* KO mice, correlational analysis between the expression levels between antibodies also failed to show a significant relationship *r*(16) = 0.41, *p* = 0.28 (Fig 4B). A lack of correlational relationships between the S499-phosphorylated FMRP expressions indicated between PhosphoSolutions^®^ and abcam^®^ antibodies in both *NS-Pten* WT and KO mice indicates that these antibodies may not be detecting changes in the same substrate.

### abcam^®^ Phosphorylated FMRP Antibody Indicates Presence of Target Protein in *Fmr-1* KO mice

**Fig 5:**
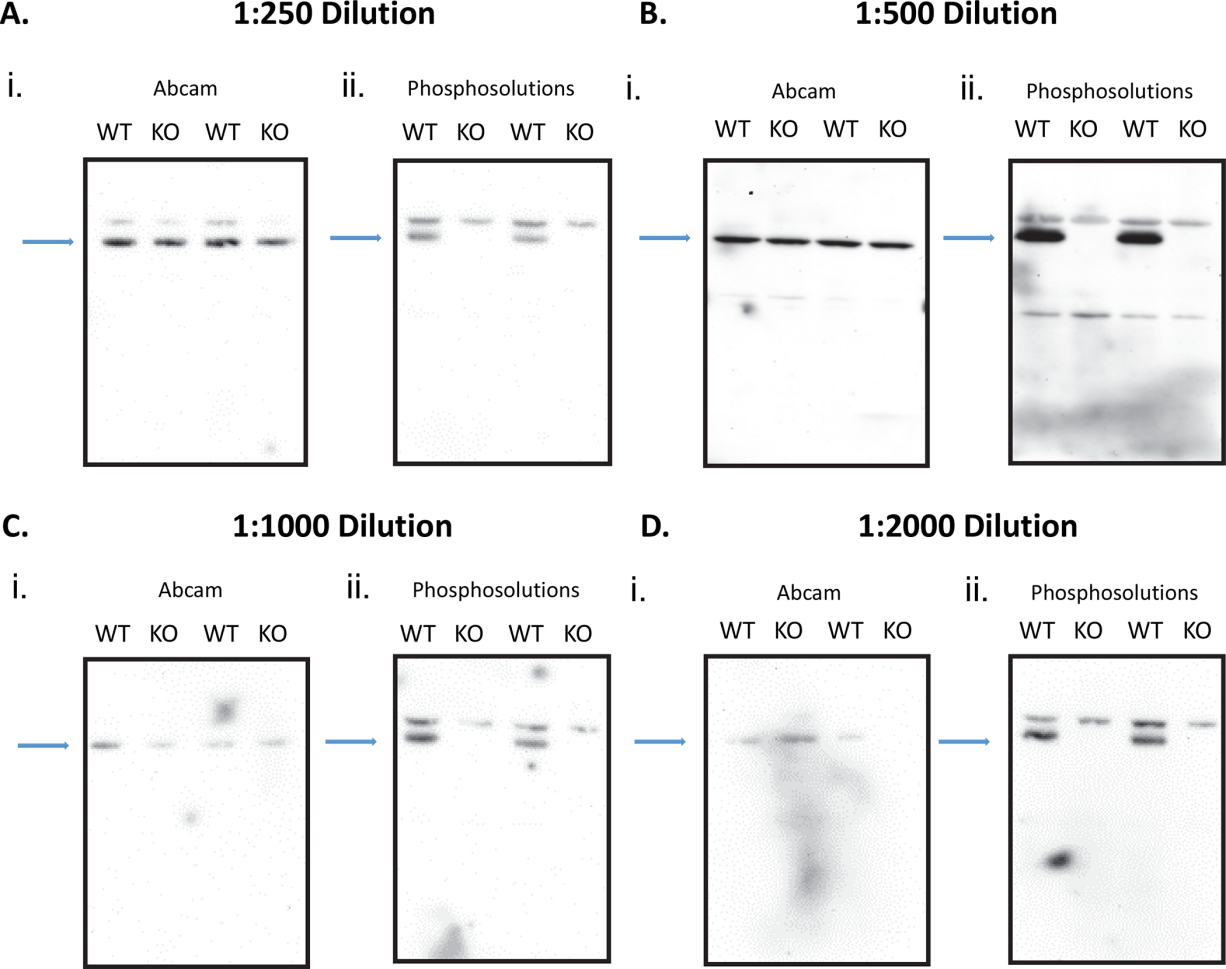
Comparison of S499-Phosphorylated FMRP Expression Between Antibodies. We examined phosphorylated levels of FMRP in *Fmr-1* wildtype (WT) and knockout (KO) mice using 4 concentrations of the abcam^®^ and PhosphoSolutions^®^ antibody. The full blot of the samples is presented in each set of figures. There are 2 alternating lanes of WT and KO probed samples throughout all dilutions. In each dilution experiment the first figure used the abcam^®^ S499 phosphorylated antibody (i) and the second figure used the PhosphoSolutions^®^ S499 phosphorylated antibody (ii). The blue arrows point to the location of the target site. (Fig 5A) WT and KO hippocampus tissue probed for antibodies using a 1:250 dilution, (Fig 5B) 1:500 dilution, (Fig 5C) 1:1000 dilution, (Fig 5D) 1:200 dilution

In order to determine whether the PhosphoSolutions^®^ and abcam^®^ antibodies are specifically targeting the S499-phosphorylated FMRP substrate, we next compared relative expression levels in *Fmr-1* KO mice. Because these mice have a congenital absence of FMRP, neither antibody should detect any presence of S499-phosphorylated FMRP. In order to rule out the nonspecific binding effects resulting from too low or high antibody concentration, we used 4 different concentrations (Fig 5A–5D). This would help to eliminate the possibility that the appearance of a band in the knockout mice is due to too high a concentration of antibody. Visual analysis of S499-phosphorylated FMRP expression using the PhosphoSolutions^®^ antibody shows an alternating pattern, indicating an absence of any expression in lanes with *Fmr-1* KO samples using a 1:250 dilution (Fig 5A), 1:500 dilution (Fig 5B), 1:1000 dilution (Fig 5C), or 1:2000 dilution (Fig 5D). In all dilutions there is a clear pattern that the PhosphoSolutions^®^ antibody correctly probes for phosphorylated FMRP, while abcam^®^ does not. Visual analysis of S499-phosphorylated FMRP expression using abcam^®^ antibody shows a constitutive expression in all lanes (Fig 5A–5D).

## Discussion

Although there are no universally accepted guidelines for validating antibodies, Bordeaux et al. (2010) recommended several steps to ensure antibody selectivity and reproducibility [23]. First, these authors advise testing antibodies against a cell line with known target expression. By comparing experimental data obtained from the antibody in question with expected values, researchers are able to determine its target selectivity. Cell lines with target gene deletion are considered to be the “Gold Standard” in antibody verification because true antibodies should not detect any measurable expression of the gene product. Bordeaux et al. (2010) also recommend verifying reproducibility of the antibody results through use of multiple molecular techniques, such as western blotting, immunoprecipitation, immunohistochemistry, or immunofluorescence.

In the present study S499-phosphorylated FMRP antibodies from PhosphoSolutions^®^ and abcam^®^ were tested against *NS-Pten* KO and *Fmr-1* KO mouse hippocampal cell lines in western blot analysis. In *NS-Pten* KO mice both antibodies correctly identified an increase in target protein expression compared to WT mice, though the abcam^®^ antibody indicated significantly greater protein expression than the antibody from PhosphoSolutions^®^. The experimental data using the abcam^®^ antibody also showed much greater variance between data points, suggesting a possible lack of specificity. Their performances continued to differ when tested in *Fmr-1* KO mice. In this cell line, only the antibody from abcam^®^ antibody identified the presence of S499-phosphorylated FMRP, which would be impossible considering that the congenital absence of genes coding for this protein. These results confirm that the abcam^®^ antibody is not selective for S499-phosphorylated FMRP.

One possible contributor to the variance in the results from the *NS-Pten* KO mice may be due to an incorrect concentration of the antibody. If the antibody concentration used was too high there could have been an increase in nonspecific binding, which would contribute to the variability seen in the phosphorylated FMRP levels in *NS-Pten* WT and KO mice. We included a series of experiments in the FMRP WT and KO mice with concentrations of abcam^®^ and PhosphoSolutions^®^ antibodies at 1:250, 1:500, 1:1000, and 1:2000 (Fig 5A–5D). A clear increase in nonspecific binding was observed in the abcam^®^ treated samples, since an additional band appeared on top of the target band when a 1:250 concentration was used (Fig 5A). However, in each of the abcam^®^ dilutions there was a consistent band seen in the knockout mice that should not be present. We did not observe this band in the FMRP KO mice when we used the Phosphosolutions^®^ antibody. Therefore, the variability seen in the *NS-Pten* KO samples using the abcam^®^ antibody was not due to nonspecific binding, but to lack of antibody specificity.

Although we did not choose to repeat this study using other molecular techniques, the reliability of the PhosphoSolutions^®^ antibody was reproduced in two different hippocampal cell lines in western blot analysis; one of which being the gold standard target gene knockout. Two previous studies have also verified this antibody by using other molecular techniques [7, 22]. Therefore, our study supports existing evidence for the selectivity and reproducibility of the PhosphoSolutions^®^ antibody, as well as informs researchers to preferentially use this product over the antibody from abcam^®^ when investigating phosphorylation changes in FMRP.

It is still unclear what the antibody from abcam^®^ might be measuring, but several possibilities exist. In a recent study by Bartley et al (2014), which validated commercially available S499-phosphorylated FMRP antibodies, it was found that one antibody also recognized unphosphorylated FMRP. These authors did not identify the company that produced this nonspecific antibody. If the antibody from abcam^®^ is measuring FMRP indiscriminant of phosphorylation status, this may explain why it indicated greater S499-phosphorylated FMRP expression than the PhosphoSolutions^®^ antibody in *NS-Pten* KO mice. However, this would not account for its identification of protein in *Fmr-1* KO mice.

It is also possible that this antibody is measuring proteins with homologous sequences to FMRP. Fragile-X Related proteins 1 and 2 (FXR1 and FXR2) are members of the same protein family as FMRP and are 70–80% homologous in the N-terminal region [24, 25]. If the antibody from abcam^®^ is measuring the FXR1/2 proteins, in addition to the total FMRP, this may explain for the protein expression it indicated in *Fmr-1* KO mice. This would also account for the large variability between its data points in *NS-Pten* KO mice.

There are several published studies that have used abcam^®^ S499-phosphorylated FMRP antibody. Given the results of this study, it may be necessary for these researchers to reproduce their findings using the PhosphoSolutions^®^ antibody in order to ensure their validity.

Antibodies are some of the most frequently used tools in basic science and clinical investigation, allowing researchers to probe specific aspects of molecular physiology. However, synthesizing these powerful tools is a highly intricate process that requires careful planning and precision. An even greater degree of difficulty is introduced when producing antibodies that recognize only specific phosphorylated amino-acid residues. Therefore, it is imperative to for researchers to validate the specificity and reproducibility of antibodies used in their investigations.
